# Level of Precompetitive Anxiety and Self-Confidence in High Level Padel Players

**DOI:** 10.3389/fpsyg.2022.890828

**Published:** 2022-06-03

**Authors:** Alfonso Castillo-Rodriguez, Antonio Hernández-Mendo, José Ramón Alvero-Cruz, Wanesa Onetti-Onetti, José Carlos Fernández-García

**Affiliations:** ^1^Department of Physical Education and Sports, University of Granada, Granada, Spain; ^2^Department of Social Psychology, Social Work, Anthropology and East Asian Studies, University of Málaga, Málaga, Spain; ^3^Department of Human Physiology, Histology, Pathological Anatomy and Sports Physical Education, University of Málaga, Málaga, Spain; ^4^Facultad de Ciencias de la Educación, Universidad Internacional De La Rioja, Logroño, Spain; ^5^Facultad de Ciencias de la Educación, Universidad de Málaga, Málaga, Spain; ^6^Andalucía-Tech, Málaga, Spain; ^7^IBIMA, Málaga, Spain

**Keywords:** anxiety, self-confidence, category, competition, raquet sports, padel

## Abstract

The objectives of the present study were firstly to evaluate precompetitive anxiety and self-confidence (SC) in padel players according to their playing level; and secondly, to study the factors that influence the levels of precompetitive anxiety and SC. One hundred padel players, all of whom were federated men (age: *M* = 27.6, SD = 7.5 years; weight: *M* = 73.4, SD = 9.8 kg; height: *M* = 175.6, SD = 7.5 cm) participated in the research. The CSAI-2 (*Competitive State Anxiety Inventory-2*) questionnaire was used and a one-way ANOVA and a two- and three-ways MANOVA were conducted. The results show that the players from a better category had higher scores in SC and lower scores in somatic anxiety (SA) (η*^2^* = 0.10 and η*^2^* = 0.12, respectively). Moreover, the factors of category, body mass index (BMI) and experience, predicted 82% of the variance explained by the SC of the player. As a conclusion, this study has made it possible to ascertain that the playing category, understood as the level of the padel players, determines the levels of anxiety and SC and represents a key factor for the prediction of sports performance.

## Introduction

Padel is a sport racket, that uses tennis rules (García-Benítez et al., 2018), played in pairs on a 20 m × 10 m court divided in two areas by a net where the enclosing walls are a part of the game (Rivilla-García et al., 2019). Matches are the best of three sets using tennis scoring (Ramón-Llin et al., 2019). Torres-Luque et al. (2015) found in elite players a mean of 9.30 s per point and 9.38 shots per point, in matches lasting 57 min. The development of these shots continuously and with such a short duration could cause a stress situation that can occur permanently prior to official competition matches (Jiménez et al., 2012).

The study of psychological factors can be of great importance for an athlete’s performance (Giske et al., 2016). Different qualities and psychological skills, like stress and anxiety control, self-confidence (SC), communication, team cohesion, motivation, mood states and, concentration, could fluctuate over time as they are not fixed and innate states, and are susceptible to improvement in time, thanks to the training of psychological skills (Guillen and Feltz, 2011) and thus, can improve sports performance (Mathers and Brodie, 2011; Slack et al., 2015; Díaz et al., 2019).

Racket sports present different physical, physiological and psychological demands, among others, due basically to the nature of play, i.e., intermittent sprints with incomplete recovery (Alvero-Cruz et al., 2009; Courel-Ibáñez et al., 2017) and constant decision-making which implies processing a large quantity of information in a short interval of time (Castillo-Rodríguez et al., 2014a), which generates high responses of anxiety and stress due to the cognitive and emotional pressure experienced.

On the one hand, anxiety in the moments prior to sports competition refers to the temporary emotional state characterized by subjective sensations of apprehension and tension, accompanied by activation of the autonomous nervous system (Martens et al., 1990). On the other hand, stress defined as a psychophysiological response of the athlete to threatening events (Jiménez et al., 2012), is triggered by the absence of adequate coping strategies that permit the redefinition of the situation as non-threatening on the part of the athlete (Alonso-Arbiol et al., 2005). These levels of anxiety and stress generated by athletes have increased due to the number of competitions that are currently organized and the high level of physical preparation thus required by the athletes to face them (Ruiz and Lorenzo, 2008), resulting in performance problems if this high level of activation is maintained.

Similarly, the SC of the athlete, which is the belief that he or she can act successfully (Robazza and Bortoli, 2007), has a positive relation with sports performance (Díaz et al., 2008), although up to an optimal point, as an excess of SC decreases these levels of performance (Weinberg and Gould, 1996). The relations between anxiety and SC and sports performance have been an object of study for decades. Thus, there is a clear consensus that indicates an inverse relation between anxiety and sports performance and SC (León-Prados et al., 2011), specifically, low levels of SC suggest higher levels of cognitive anxiety (CA) (Pinto and Vázquez, 2013) and a high level of anxiety suggests a lower sports performance (García-Mas et al., 2011; Ries et al., 2012), which leads us to consider the initial hypothesis that athletes with a better sports performance, who play in higher categories, possess lower levels of anxiety and higher ones of SC than athletes in lower categories.

Martens et al. (1990) developed an instrument to analyze the constructs related to anxiety and SC called the Competitive State Anxiety Inventory-2 (CSAI-2), which was identified as one of the most commonly used tools for analyzing these constructs (Morillo Baro et al., 2016). CA is characterized by an understanding of the negative expectations and cognitive worries that a person has about him or herself and their environment; and somatic anxiety (SA) is manifested through signs of physiological activation, e.g., degree of muscle tension, and increase in heart rate, among others (Andrade Fernández et al., 2007).

In racket sports, it was found that tennis players from a higher category showed a strong inverse relation with precompetitive anxiety (Ryska, 1998), and moreover, the tennis players that won official competitions, had presented lower levels of CA and SA than those who eventually lost (Covassin and Pero, 2004). However, in spite of these investigations, there is no scientific evidence from the study of the responses of anxiety and SC prior to sports competition in padel players. Recently, a study has described stress control in padel players between 20 and 35 years of age without specifying their category or skill level (Almendros-Pacheco et al., 2022). Therefore, the objectives of the present study were first to assess the states of anxiety and SC prior to competition in padel players, bearing in mind their playing category; and secondly to study the factors that influence the levels of precompetitive anxiety and SC.

## Materials and Methods

### Participants

One hundred athletes participated in this study. Their characteristics can be seen in Table 1. They belonged to different playing categories (C1, C2, and C3), according to the structural composition of teams proposed by the Spanish Padel Federation. Most of the participants were Spanish, except 4 who were Argentinian. All the championships evaluated were of a national nature. Different categories were added according to the category usually played by the subjects during the last year. All players were informed about the characteristics and objectives of the study and signed an informed consent form. The research was conducted according to the indications established in the Declaration of Helsinki (2013) and the Ethics Committee of the University of Granada gave its approval (Ref. n° 471/CEIH/2018).

**TABLE 1:** Characteristics of padel players depending on the category.

		C1 (*N* = 26)	C2 (*N* = 16)	C3 (*N* = 20)	*P*	η*^2^*
Age	(years)	27.96 ± 6.16	27.11 ± 9.39	27.97 ± 6.98	0.768	0.05
Weight	(kg)	78.62 ± 10.0**^2^**	68.10 ± 8.02**^1^**	73.44 ± 11.5	0.001	0.27
Height	(cm)	176.0 ± 7.45	174.0 ± 7.65	176.7 ± 7.24	0.465	0.09
BMI	(kg⋅m^–2^)	25.35 ± 2.49**^2,3^**	22.48 ± 2.08**^1^**	23.48 ± 2.89**^1^**	0.001	0.31
GT	(hours/week)	2.60 ± 3.05	1.78 ± 3.17	1.21 ± 1.65	0.068	0.16
ST	(hours/week)	14.44 ± 13.4**^3^**	13.33 ± 13.1	7.15 ± 8.81**^1^**	0.025	0.19
TT	(hours/week)	17.04 ± 14.3**^3^**	15.11 ± 12.2	8.36 ± 9.00**^1^**	0.008	0.22
Experience	(years)	6.52 ± 4.04**^3^**	6.78 ± 5.03**^3^**	4.00 ± 2.47**^1,2^**	0.007	0.22

*C1: players of highest category; C2: players of moderate category 2; C3: players of lowest category. ANOVA test (P < 0.05); Bonferroni Post hoc test: ^1^Differences with C1; ^2^Differences with C2; ^3^Differences with C3; GT, General training; ST, Specific training; TT, Total training; Experience: Experience of padel.*

### Instruments

Weight and height were assessed through a wall-mounted stadiometer (Seca 220, Hamburg, Germany), and a calibrated electronic digital scale (Seca 769, Hamburg, Germany), respectively. Players were analyzed on the match day because they came from different Spanish cities, corresponding to their usual place of residence. Therefore, all of them did not come fasting, but all of them had eaten juice and bread at least 2 h before the competition.

The CSAI-2 (*Competitive State Anxiety Inventory-2*) questionnaire by Martens et al. (1990) comprising 27 items with a Likert scale of 1–4, was administered. This questionnaire assesses the two basic components of anxiety. The first is the state of CA or worry about a possible failure and the adverse consequences that derive from it, which manifest as negative thoughts, restlessness and doubt, negative expectations and loss of concentration. The second component refers to the state of SA or elevation of the level of activation of physiological functions (heart rate, respiration, and muscle tension, among other symptoms) which produce nervousness and tension. As well as the cognitive and somatic components, a third component is identified, SC, which is conceptually similar to perceived self-sufficiency. In this study, Cronbach’s alpha has been analyzed in the psychological variables (CA: 0.702; SA: 0.771; SC: 0.839).

### Procedure

The participants in this research were selected in an intentional and stratified manner, i.e., the possible participants were divided into categories and a small number of cases were selected to be studied in depth in each competition. These championships were held in Spanish cities with altitudes varying between 15 and 106 m above sea level. The environmental conditions were very similar in all the matches analyzed, with a temperature of between 19–23°C and between 40 and 56% humidity. The questionnaire was administered to the players 60 min before the start of the match. Moreover, demographical data was also recorded like years of experience, general training hours per week, padel training hours per week and playing category. Measurements were subsequently obtained for weight, height and age, and body mass index (BMI) was calculated.

### Statistical Analysis

The statistical analyses were performed with *SPSS for Windows v.20.0* (*SPSS Inc*., Chicago, IL, United States). The normality of the variables was assessed with the Kolmogorov-Smirnov test and the size of the sample was calculated with an α value of 0.05. Descriptive and comparative analyses were then conducted with the category factor with 3 levels (ANOVA and Kruskal Wallis for non-normal variables) and correlations (Pearson’s coefficient and Spearman’s Rho for non-normal variables). Additionally, a two-way (category-experience and experience-BMI) MANOVA (multiple analysis of variance) and a three-way (category-experience-BMI) MANOVA were performed. Effect size (η2) was also analyzed which quantified the size of the difference that exists between both groups. According to this, we could say that this is a true measure of the importance of the said difference (Coe and Merino, 2003). The threshold values for the Cohen effects sizes in the ANOVA test were small: 0.10; moderate, 0.25; and large, 0.40 (Cohen, 1988). The *post hoc* analysis was adjusted with the Bonferroni test and the accepted significance level was *P* < 0.05.

## Results

**FIGURE 1:**
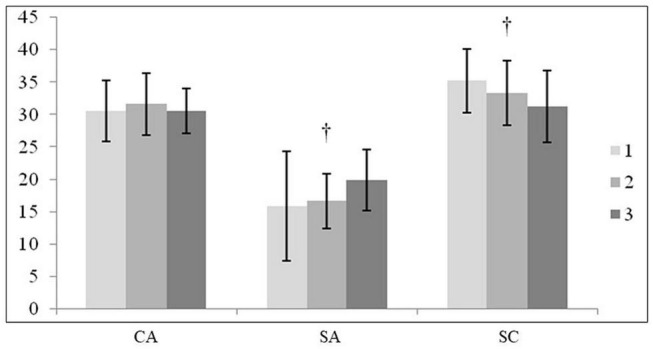
Differences in the anxiety and self-confidence variables between the 3 game categories. ^†^: Indicates differences between categories 1 and 3. 1: Category 1; 2: Category 2; 3: Category 3; CA, cognitive anxiety; SA, somatic anxiety; SC: self-confidence.

The results found using the one-way ANOVA indicated significant differences between C1 and C3 in the responses to SA and SC (Figure 1). A lineal decrease in SA can be observed according to the playing category [*F*_(1,97)_ = 7.888; *P* < 0.01; η*^2^* = 0.12]. With regard to SC, the players at the C3 level scored 31.29 ± 5.6 points and those at the C1 level 35.25 ± 4.9 points [*F*_(1,97)_ = 5.474; *P* < 0.01; η*^2^* = 0.10].

The correlations between the psychological variables according to the playing category were then analyzed (Table 2). These correlations differ among the categories, although SA and SC showed mean negative relations in all the categories (*r* = –0.34 and –0.54; *P* < 0.05).

**TABLE 2:** Correlation coefficient between psychological variables.

	SA	SC
High level (C1)		
CA	0.72**	–0.26
SA		–0.34*
Moderate level (C2)		
CA	0.10	0.21
SA		–0.54**
Low level (C3)		
CA	0.27	–0.10
SA		–0.45**

*P < 0.05; **P < 0.01; CA, cognitive anxiety; SA, somatic anxiety; SC, self-confidence.

With respect to the relation between the psychological variables and the category and training, an inverse relation was found between experience and category (rho = –0.26, *P* < 0.01) and SC was found to relate to this experience and category (*r* = 0.21 and –0.30; *P* < 0.05; respectively; Table 3).

**TABLE 3:** Relationship between psychological variables and category, experience, BMI and specific training.

	Cat	CA	SA	SC
Experience	–0.26**	0.05*	0.01**	0.21**
Cat		–0.01*	0.38**	–0.30**
BMI		–0.21*	–0.34**	0.17**
ST		–0.12*	–0.19**	0.02**

*P < 0.05; **P < 0.01; Cat, Category; CA, cognitive anxiety; SA, somatic anxiety; SC, self-confidence; ST, specific training (raquet).

Finally, CA was influenced by the experience and category of the player [*F*_(1,23)_ = 2.189, *P* = 0.02, η*^2^_*p*_* = 0.42], so that it was greater in more experienced players. Moreover, SC was influenced by a significant interaction between the factors Category-Experience-IMC [*F*_(1,22)_ = 11.170, *P* = 0.001, η*^2^_*p*_* = 0.60]. The *post hoc* Bonferroni comparison indicated high values of SC in C1 with regard to C3 (*P* < 0.001). Finally, SA was influenced by the factors experience-BMI [*F*_(1,22)_ = 3.600, *P* = 0.01, η*^2^_*p*_* = 0.45], in which SA was lower when BMI was higher.

## Discussion

The objectives of this study were firstly, to assess CA, SA, and SC prior to sports competition in padel players according to playing category; and secondly, to analyze the factors that influence the levels of precompetitive anxiety and SC. As an initial hypothesis it was established that the players from a higher level or category would score higher values in SC and lower values in CA and SA. This hypothesis was accepted in part, as the results indicated that the levels of SA were lower and those of SC higher in the padel players who participate in the highest category. This may be due to the fact that the SC of an athlete increases as they accumulated victories in competition, as determined by Weinberg and Gould (1996). Thus, the higher the number of wins, the greater the probability of playing in a higher category the following season. The 80% of the points scored in padel were carried-out at the net (Torres-Luque et al., 2015), using the volley technique. This fact allows us to know that these players increase their SC during the game and after it (Muñoz et al., 2017).

These results between the playing categories could be compared with other studies in the sport of tennis, as until now, there have been no studies on this topic in padel. Lundqvist and Hassmén (2005) indicated that high level tennis players scored lower values of SC with 31.1 ± 7.6 points, compared to the padel players. However, they confirmed their superiority with respect to players from C3 with 28.4 ± 7.5 points, they were also considerably below those found in padel, also showing significant differences between these categories. Although the CSAI-2 test was administered at the same time before the competition (60 min), the difference in scores could be due to the lesser experience of these tennis players. It has been shown that SC is affected by the category, experience, and BMI factors. Moreover, in this same sport of tennis, studies both in players from C1 by Cervelló et al. (2002) and Letelier (2007), and in players from C3 by Ryska (1998) showed a clear difference in SC compared to those of padel (26.0 ± 8.0; 27.9 ± 4.5, and 24.69 ± 6.6, respectively). Only in the study by Covassin and Pero (2004) had a similarity been confirmed in the responses on SC, in tennis players from C2 (32.0 ± 3.6 points), using in all cases the same instrument (CSAI-2). Finally, we could state that SC may be an important factor for differentiating elite and non-elite athletes, as also indicated in the study by Li et al. (1999). This SC was explained in up to 82% of the variance through the category, experience, and BMI factors.

With regard to anxiety, the playing category more than experience has a strong inverse relation with precompetitive anxiety (Ryska, 1998). This study showed a clear difference between the players from C1 and C3 (*P* < 0.05; Figure 1). However, both CA and SA were influenced by experience and BMI. On the one hand, CA was higher in experienced players. The player’s experience is proportional to the expectation of success and thus corresponds to a greater manifestation of concern (response of CA). Thus, it is recommended to include a program for training psychological skills to attenuate this high level of CA (Mathers and Brodie, 2011; Slack et al., 2015). On the other hand, SA was lower when the BMI is higher, basically because the players with more muscle mass are those with a greater BMI and thus, less anxiety. An inverse relation has been found between specific training and SA, which denotes more muscle mass.

Playing category was related to SA which was lower in higher categories (*P* < 0.05), confirming that the players from C1 showed less SA than those from C3. These results were similar to those found in the study by García et al. (2009), who observed that the scores for precompetitive anxiety gradually diminish (*P* < 0.05), as the playing level or category increases (regional, national, and international). This difference in SA may be due to different factors like for example the player’s self-concept, age, experience, and morphology, in which a greater mesomorphic component stood out in players from C1 (Castillo-Rodríguez et al., 2014b). Furthermore, the higher the player’s level, the more distance was covered at lower speeds that may be due to efficiency in the first category players’ movements (Mellado-Arbelo and Baiget, 2022). This efficiency in highest level players, could be the cause of having the lowest SA showed in this study. In contrast, in golf, an inverse progressive increase was evident, that is, the higher the playing level, the greater the levels of anxiety, due to the physiological and psychological requirements characteristic of this sport (Chamberlain and Hale, 2007).

This study has some strengths. First, it is the first study to analyze anxiety in padel players prior to official competition. Secondly, it has been shown that the padel players of higher category or expertise, are those who have less anxiety and greater SC before a padel match. Finally, it has been shown that age, experience and BMI are factors that predict 80% of the variance explained, the SC of the padel player. However, the present study has several limitations. Due to the decrease in statistical power (effect size) the women players were excluded from the initial database. For future studies, researchers are encouraged to assess if behavior regarding anxiety and SC is similar in both genders. Moreover, it is recommended to record the final result of the matches, to analyze if in the end precompetitive anxiety influences the victory or defeat of the players. In the present study, the fat and muscle mass of the players have not been analyzed, which would be interesting to contrast that the players with the highest BMI are those with the greatest muscle mass, and even be able to make more rigorous statements regarding to these anthropometric variables. The authors consider that the BMI value in performance should not be treated in isolation.

To conclude, levels of anxiety and SC prior to sports competition have been described in padel players according to playing category. The main findings show that the levels of SC increase linearly as the player progresses to a higher category and this construct is predicted by the factors of category-BMI-experience in 82% of the explained variance. Specifically, category represents 28.4% of the explained variance in precompetitive SC in padel players. Lastly SA decreases linearly the higher the category of the padel player, confirming that players from lower categories could include intervention programs to improve psychological skills with the aim of facing competition with fewer psychological states related to anxiety, in the same way as they are recommended for players in the highest category to avoid dropping to a lower one.

## Data Availability Statement

The raw data supporting the conclusions of this article will be made available by the authors, without undue reservation.

## Author Contributions

AC-R, JF-G, AH-M, and JA-C contributed to the conception and design of the study. AC-R and WO-O organized the database. AC-R, JF-G, and JA-C performed the statistical analysis. AC-R, WO-O, and JA-G wrote the first draft of the manuscript. All authors wrote sections of the manuscript, and contributed to manuscript revision, read, and approved the submitted version.

## Conflict of Interest

The authors declare that the research was conducted in the absence of any commercial or financial relationships that could be construed as a potential conflict of interest.

## Publisher’s Note

All claims expressed in this article are solely those of the authors and do not necessarily represent those of their affiliated organizations, or those of the publisher, the editors and the reviewers. Any product that may be evaluated in this article, or claim that may be made by its manufacturer, is not guaranteed or endorsed by the publisher.

## References

[B1] Almendros-Pacheco J. Moral-García J. E. Arroyo-Del Bosque R. Maneiro R. (2022). Análisis de las características psicológicas relacionadas con el rendimiento deportivo en pádel. *Logía Educ. Física Deporte* 2 46–57.

[B2] Alonso-Arbiol I. Falcó F. López M. Ordaz B. Ramírez A. (2005). Development of a questionnaire for the assessment of sources of stress in Spanish soccer referees. *Ansiedad Estrés* 11 175–188.

[B3] Alvero-Cruz J. R. Barrera J. Mesa A. Cabello Manrique D. (2009). “Correlations of physiological responses in squash players during competition,” in *Science and Racket Sports IV*, eds Lees A. Cabello Manrique D. Torres G. (Oxon: Routledge), 64–69.

[B4] Andrade Fernández E. M. Lois Río G. Arce Fernández C. (2007). Propiedades psicométricas de la versión española del Inventario de Ansiedad Competitiva CSAI-2R en deportistas. *Psicothema* 19, 150–155. 17295997

[B5] Castillo-Rodríguez A. Alvero-Cruz J. R. Hernández-Mendo A. Fernández-García J. C. (2014a). Physical and physiological responses in Paddle Tennis competition. *Int. J. Perform. Anal. Sport* 14 524–534. 10.1080/24748668.2014.11868740

[B6] Castillo-Rodríguez A. Hernández-Mendo A. Alvero-Cruz J. R. (2014b). Morphology of the Elite Paddle Player. Comparison with other Racket Sports. *Int. J. Morphol.* 32 177–182. 10.4067/S0717-95022014000100030 27315006

[B7] Cervelló E. Santos-Rosa F. J. Jiménez R. Nerea A. García T. (2002). Motivación y ansiedad en jugadores de tenis. *Eur. J. Hum. Move.* 9 141–161.

[B8] Chamberlain S. T. Hale B. D. (2007). Competitive state anxiety and self-confidence: intensity and direction as relative predictors of performance on a golf putting task. *Anxiety Stress Coping* 20 197–207. 10.1080/10615800701288572 17999224

[B9] Coe R. Merino C. (2003). Magnitud del efecto: una guía para investigadores y usuarios. *Rev. Psicol.* 21 147–177. 10.18800/psico.200301.006

[B10] Cohen J. (1988). *Statistical Power Analysis for the Behavioral Sciences*, 2nd Edn. Mahwah, NJ: Lawrence Erlbaum Associates.

[B11] Courel-Ibáñez J. Sánchez-Alcaraz B. J. García S. Echegaray M. (2017). Evolution of padel in Spain according to practitioners’ gender and age. *Cult. Cienc. Deporte* 12 39–46. 10.12800/ccd.v12i34.830

[B12] Covassin T. Pero S. (2004). The relationship between self-confidence, mood state, and anxiety among collegiate tennis players. *J. Sport Behav.* 27 230–242.

[B13] Díaz E. M. Rubio S. Martín J. Luceño L. (2008). Rendimiento deportivo en jugadoras de élite de hockey hierba: diferencias en ansiedad y estrategias cognitivas. *Rev. Psicol. Educ.* 7 23–41.

[B14] Díaz J. Muñoz D. Cordero J. Robles M. Courel-Ibáñez J. Sánchez-Alcaraz B. (2019). Estado de ánimo y calidad de vida en mujeres adultas practicantes de pádel. *Rev. Iberoam. Cienc. Actividad Física Deporte* 7 34–43. 10.24310/riccafd.2018.v7i3.5538

[B15] García V. Ruiz L. M. Graupera J. L. (2009). Perfiles decisionales de jugadores y jugadoras de voleibol de diferente nivel de pericia. *Rev. Int. Cienc. Deporte* 14 123–137. 10.5232/ricyde2009.01410

[B16] García-Benítez S. Courel-Ibáñez J. Pérez-Bilbao T. Felipe J. L. (2018). Game responses during young padel match play: age and sex comparisons. *J. Strength Cond. Res.* 32 1144–1149. 10.1519/JSC.0000000000001951 29112057

[B17] García-Mas A. Palou P. Smith R. E. Ponseti X. Almeida P. Lameiras J. (2011). Ansiedad competitiva y clima motivacional en jóvenes futbolistas de competición, en relación con las habilidades y el rendimiento percibido por sus entrenadores. *Rev. Psicol. Deporte* 20 197–207.

[B18] Giske R. Hausen T. Johansen B. T. (2016). Training, mental preparation, and unmediated practice among soccer referees: an analysis of elite and sub-elite referees’ reported practice. *Int. J. Appl. Sport Sci.* 28 31–41. 10.24985/ijass.2016.28.1.31

[B19] Guillen F. Feltz D. L. (2011). A conceptual model of referee efficacy. *Front. Psychol.* 2:25. 10.3389/fpsyg.2011.00025 21713174 PMC3111226

[B20] Jiménez M. Aguilar R. Alvero-Cruz J. R. (2012). Effects of victory and defeat on testosterone and cortisol response to competition: evidence for same response patterns in men and women. *Psychoneuroendocrinology* 37 1577–1581. 10.1016/j.psyneuen.2012.02.011 22429747

[B21] León-Prados J. A. Fuentes I. Calvo A. (2011). Ansiedad estado y autoconfianza precompetitiva en gimnastas. *Rev. Int. Cienc. Deporte* 22 76–91. 10.5232/ricyde2011.02301

[B22] Letelier A. (2007). *Estudio Correlacional Entre la Ansiedad Estado Competitiva y las Estrategias de Afrontamiento Deportivo en Tenistas Juveniles.* Santiago: Universidad de Chile.

[B23] Li N. Fan Y. Guan X. Zhao M. (1999). *Research of Pre-Competitive Anxiety of Shooting Athletes. National Sport Information Centre, Australian Sports Commission, Sports Medicine Australia. 31st October – 5th November, the 5th Word Congress on Sport Sciences.* Sydney: International Olympic Committee.

[B24] Lundqvist C. Hassmén P. (2005). Competitive State Anxiety Inventory-2 (CSAI-2): evaluating the Swedish version by confirmatory factor analyses. *J. Sports Sci.* 23 727–736. 10.1080/02640410400021484 16195023

[B25] Martens R. Burton D. Vealey R. S. Bump L. A. Smith D. E. (1990). “Development and validation of the competitive state anxiety inventory-2,” in *Competitive Anxiety in Sport*, eds Martens R. Vealey R. S. Burton D. (Champaign: Human Kinetics), 117–190.

[B26] Mathers J. F. Brodie K. (2011). Elite refereeing in professional soccer: a case study of mental skill support. *J. Sport Psychol. Action* 2 171–182. 10.1080/21520704.2011.609018

[B27] Mellado-Arbelo O. Baiget E. (2022). Activity profile and physiological demand of padel match play: a systematic review. *Kinesiology* 54 51–61. 10.26582/k.54.1.6

[B28] Morillo Baro J. P. Garrido R. Enrique R. Hernández-Mendo A. (2016). Relaciones entre el perfil psicológico deportivo y la ansiedad competitiva en jugadores de balonmano playa. *Rev. Psicol. Deporte* 25 121–128.

[B29] Muñoz D. Courel-Ibáñez J. Sánchez-Alcaraz B. J. Díaz J. Grijota F. J. Muñoz J. (2017). Análisis del uso y eficacia del globo para recuperar la red en función del contexto de juego en pádel. *Retos* 31 19–22.

[B30] Pinto M. F. Vázquez N. (2013). Ansiedad estado competitiva y estrategias de afrontamiento: su relación con el rendimiento en una muestra argentina de jugadores amateurs de golf. *Rev. Psicol. Deporte* 22 47–52.

[B31] Ramón-Llin J. Guzmán J. F. Llana S. Martínez-Gallego R. James N. Vučković G. (2019). The effect of the return of serve on the server pair’s movement parameters and rally outcome in padel using cluster analysis. *Front. Psychol.* 10:1194. 10.3389/fpsyg.2019.01194 31191397 PMC6546820

[B32] Ries F. Castañeda C. Campos M. C. del Castillo O. (2012). Relaciones entre ansiedad-rasgo y ansiedad-estado en competiciones deportivas. *Cuad. Psicol. Deporte* 12 9–16. 10.4321/S1578-84232012000200002

[B33] Rivilla-García J. Muñoz Moreno A. Lorenzo J. Van den Tillaar R. Navandar A. (2019). Influence of the opposition on overhead smash velocity in padel players. *Kinesiology* 51 206–212. 10.26582/k.51.2.6

[B34] Robazza C. Bortoli L. (2007). Perceived impact of anger and anxiety on sporting performance in rugby players. *Psychol. Sport Exerc.* 8 875–896. 10.1016/j.psychsport.2006.07.005

[B35] Ruiz R. Lorenzo O. (2008). Características psicológicas en los jugadores de pádel de alto rendimiento. *Rev. Iberoam. Psicol. Ejercicio Deporte* 3 183–200.

[B36] Ryska T. A. (1998). Cognitive-behavioral strategies and precompetitive anxiety among recreational athletes. *Psychol. Record* 48 697–708. 10.1007/BF03395299

[B37] Slack L. A. Maynard I. W. Butt J. Olusoga P. (2015). An evaluation of a mental toughness education and training program for early-career English football league referees. *Sport Psychol.* 29 237–257. 10.1123/tsp.2014-0015

[B38] Torres-Luque G. Ramírez A. Cabello-Manrique D. Nikolaidis P. Alvero-Cruz J. (2015). Match analysis of elite players during paddle tennis competition. *Int. J. Perform. Anal. Sport* 15 1135–1144. 10.1080/24748668.2015.11868857

[B39] Weinberg R. S. Gould D. (1996). *Fundamentos de Psicología del Deporte y Ejercicio Físico.* Barcelona: Ariel.

